# Negative Correlation of LIV-1 and E-Cadherin Expression in Hepatocellular Carcinoma Cells

**DOI:** 10.1371/journal.pone.0056542

**Published:** 2013-02-20

**Authors:** Rongxi Shen, Feng Xie, Hui Shen, Qu liu, Tao Zheng, Xingrui Kou, Dexian Wang, Jiamei Yang

**Affiliations:** 1 Department of Special Treatment, Eastern Hepatobiliary Surgery Hospital, Second Military Medical University, Shanghai, China; 2 Department of Military Hygiene, Second Military Medical University, Shanghai, China; 3 Tumor Immunology and Gene Therapy Center, Eastern Hepatobiliary Surgery Hospital, the Second Military Medical University, Shanghai, China; The University of Hong Kong, China

## Abstract

LIV-1, a zinc transporter, is a mediator downstream of STAT3 both in zebrafish and mammalian cells, and is involved in epithelial-mesenchymal transition (EMT). Despite LIV-1 participates in cancer growth and metastasis, little is known about the association of LIV-1 with human liver cancer development. Therefore, the expression of LIV-1 mRNA was analyzed by reverse transcriptase polymerase chain reaction (RT-PCR) in 4 cultured cell lines (3 carcinoma and 1 normal liver cell lines), and the localization of LIV-1 protein was investigated by immunohistochemistry. Expression of LIV-1 protein was analyzed by Western blot both in 4 cultured cell lines and 120 liver tissues (100 carcinoma and 20 histologically normal tissues), and the relationship between its expression and clinicopathological finding was investigated in 100 hepatocellular carcinoma(HCC) tissues. Then stable siRNA expressing Hep-G2 cells were generated to assess the function of LIV-1 in liver cancer cells. We found that LIV-1 mRNA was more highly expressed in liver cancer cell lines compared to normal liver cell line. Western blot showed the expression of LIV-1 was higher in 61% liver carcinoma tissues than that in normal liver tissues. Down-regulated LIV-1 cells showed significant inhibition of proliferation in vitro and reduction of tumor growth in vivo. Furthermore, E-cadherin expression increased in LIV-1 siRNA expressing Hep-G2. These findings indicated that LIV-1 may induce the EMT in HCC cells.

## Introduction

LIV-1 has been originally identified as a member of new subfamily of zinc transporters, termed LZT (LIV-1 subfamily of ZIP zinc transporters) and suggested to be located to the plasma membrane. As a zinc transporter, LIV-1 transports zinc into cells [1]. Zinc is essential for life and, as such, is involved in the control of gene transcription, differentiation, development and growth [2], [3], suggesting that its altered distribution might promote tumorigenesis. LIV-1 was early identified as a gene whose expression was stimulated by oestrogen in the breast cancer cell line ZR-75 [4] and showed a highly significant association with the spread of breast cancer to the regional lymph nodes [5].

Epithelial-mesenchymal transition (EMT) is one of central events in embryonic development, tissue remodelling and wound repair. This transition is also considered to be important in malignant tumor progression and metastasis [6], [7]. As a repressor of EMT, E-cadherin is a major component of adherens junctions and its alterations in expression or function occur frequently in both embryogenesis and carcinogenesis, in which its loss can lead to tumor cell migration and invasion [8]. Previous study revealed that LIV-1 was involved in EMT of gastrula organizer cells in zebrafish by regulating Snail, which has been shown to be master regulator of EMT through down-regulation of E-cadherin [9]. In addition, LIV-1 was overexpressed in cervical cancer and LIV-1 suppression inhibited HeLa cell invasion through targeting MAPK-mediated Snail and Slug expression [10], suggesting that LIV-1 facilitates carcinoma cell invasion and metastasis.

Liver cancer is the sixth most common incident cancer and the third most common cause of cancer death [11], [12]. And the pathogenic mechanisms regulating the aggressive behavior of this cancer need to be further studied. EMT is the possible mechanism in accelerating invasion or metastasis for liver cancer cells. Little is known about LIV-1 expression and its association with EMT in liver cancer. Therefore, we tried to assess the correlation between LIV-1 and E-cadherin expression in human liver cancer and the effect of LIV-1 expression on the cell growth to explore the possible mechanisms associated with the aggressive behavior of liver cancer cells. LIV-1 could be an attractive new therapeutic target for the inhibition of liver cancer EMT and tissue metastases.

## Materials and Methods

### Cell Lines and Cell Culture

The following 3 liver cancer cell lines (SMMC-7721, Hep-3B and Hep-G2) and 1 normal liver cell line(L02) were employed in this study. Cells were purchased from cell bank of the Chinese Academy of Sciences and cultured in DMEM(Invitrogen) supplemented with 10% FBS at 37°C with 5% CO_2_ in a humidified environment.

### siRNA Knockdown and Transfection

LIV-1 siRNA was purchased from Invitrogen. Hep-G2 cells were seeded at 3×10^5^ cells per well in 6-well plates for 24 hours. The cells were transfected with 2.5 ml of 20 mM LIV-1 siRNA or equal amount of universal control siRNA, using 8 ml Lipofectamine 2000 (Invitrogen) per well. Cells were harvested and assayed 48 hours after transfection.

### RNA Extraction and Reverse Transcription-polymerase Chain Reaction (RT-PCR)

Total RNA from the transfected or non-transfected cells was extracted at 48 h post-transfection using Trizol Reagent (Invitrogen) and then 1 mg RNA was used in first–strand cDNA synthesis reaction with the Superscript First-Strand cDNA Synthesis kit (Invitrogen).Equal volume of cDNA (3 µl) from each reaction were used for PCR analysis.

The following primers were used: 5′-GAAATCCCTCCAAAGACCTAT-3′ and 5′-ATGACTATGGTGGTGACTTGC-3′for LIV-1; 5′-GGAATCCAAAGCCTCAGGTCAT-3′ and 5′-GGCAGTAAGGGCTCTTTGACC-3′ for E-cadherin; 5′-CGAAAGGCCTTCAACTGCAAAT-3′ and 5′-ACTGGTACTTCTTGACATCTG-3′ for Snail; 5′-GTGAAGGTCGGAGTCAACGG-3′ and 5′-CCTGGAAGATGGTGATGGGAT-3′ for GAPDH, which was used as an internal control.

The cDNA was amplified for 32 (LIV-1, E-cadherin, snail) and 28 cycles (GAPDH), using the following parameters: 94°C for 30 s, 52°C (LIV-1), 58°C (E-cadherin) and 59°C (GAPDH) for 30 s and 72°C for 30 s, with a final extension step at 72°C for 10 min.PCR products were electrophoresed through 1.5% agarose gel, stained with ethidium bromide (EB) and visualized under ultraviolet illumination. Band intensity was calculated densitometrically using Quantity One-4.4.1 imaging software (Bio-Rad Laboratories). Levels of mRNA were expressed as the ratio of band intensity for LIV-1 or E-cadherin relative to that for GAPDH.

### Immunohistochemistry (IHC)

To investigate location of the LIV-1 protein, HCC tissues were formalin-fixed and embedded in paraffin. 5-µm sections were prepared. After blocking endogenous peroxidase activity and non-specific staining, the sections were incubated overnight at 4°C with the rabbit SLC39A6 (LIV-1) antibody (1∶200, Abcam, UK) overnight at 4°C. HRP-labeled goat anti-mouse immunoglobulin (1∶100, Santa Cruz Biotechnology, Inc. Cat. SC-2005) was applied as secondary antibody for 40 min at 37°C. Immunostaining was developed using DAKO Liquid DAB+ Substrate-Chromogen System (ZSJQ Biotechnology, Beijing, China), followed by counterstaining with hematoxylin. Image of tumor tissue was taken by using LEICA DMRXA2 microscope.

### Western Blot Analysis

Tissues lysates were obtained by manual agitation and lysed in RIPA lysis buffer (Beyotime, China) with 1 mM PMSF, the transfected or non-transfected cellswere also lysed in RIPA lysis buffer (Beyotime, China) with 1 mM PMSF after the designated treatments. Equal amounts of total proteins were separated by 10% sodium dodecyl sulfatepolyacrylamide gel electrophoresis (SDS-PAGE) and following the protocol suggested by the manufacturer (Bio-Rad). After blocking with 5% nonfat dry milk in TBS containing 0.2% Tween 20, membranes were incubated at 4°C overnight with anti-LIV-1 rabbit polyclonal antibody (1∶500 dilution) and anti-E-cadherin rabbit polyclonal antibody (Boster, 1∶500 dilution) primary antibodies, developed with the BeyoECL Plus substrate system (Beyotime, China, Cat. P0018). Blots were stripped and re-probed with anti-GAPDH (Epitomics, Inc. Cat. #2251-1) to confirm equal protein loading.

### Tissue Samples

The study was approved by the Committee on Ethics, the third affiliated hospital of the Second Military Medical University, informed consent which has been conducted according to the principles expressed in the Declaration of Helsinki was obtained from each patient. All participants provided their written informed consent to participate in this study. HCC tissues were obtained from patients who underwent surgical operations for the tumors at Eastern Hepatobiliary Surgery Hospital. All patients suffer from hepatitis B. The tissues collected during surgery were immediately snap-frozen in liquid nitrogen and stored at −80°C until fixed in 10% paraformaldehyde overnight and embedded in paraffin wax. LIV-1 protein was obtained from frozen tissue samples (100 HCC and 20 histologically normal livers). RT-PCR, IHC and Western blot were performed as described above. The level of target gene expression in each sample was normalized to the respective GAPDH expression level.

### MTT Assay

Cell proliferation of tranfected or non-tranfected Hep-G2 cells was measured by 3-(4,5-dimethylthiazol-2-yl)-2,5-diphenyl-2H-tetrazolium bromide (MTT) assay. Briefly, cells were seeded into 96-well plates, and on the day of harvest, 100 µl of spent medium was replaced with an equal volume of fresh medium containing 10% MTT 5 mg/ml stock. Plates were incubated at 37°C for 4 h and shaken at room temperature for 10 min. Cellular viability was determined by measuring the absorbance of the converted dye at a wavelength of 570 nm using an enzyme-linked immunosorbent assay reader (Bio-rad Inc, USA).

### Tumor Growth in Nude Mice

Tumor formation in vivo was assayed in male athymic BALB/c nude mice (5-week-old) by subcutaneously injecting each of 2×10^7^ cells suspended in 100 µl sterile PBS. Tumor volume was measured every week after the first incidence of tumor formation. Volume was determined by the equation V = L×W^2^×0.5 where V is volume, L is length and W is width. The mice were sacrificed 32 days after injection and confirming the histology by H&E staining. All animal studies were approved by the Committee on Ethics, the third affiliated hospital of the Second Military Medical University and conducted in accordance with the National Institutes of Health guidelines for the use of laboratory animals.

### Statistical Analysis

Statisticians at the Department of Statistics of the Second Military Medical University (Shanghai, China) performed the statistical analyses using the SPSS version 18.0 software package(SPSS, Chicago, IL, USA). The chi-squared test was used to analyze the correlation between LIV-1 expression and clinicopathological findings. The difference between two groups was statistically analyzed by t-test.The criterion for statistical significance was taken as *P*<0.05.

## Results

### LIV-1 Expression in Liver Cancer

**Figure 1:**
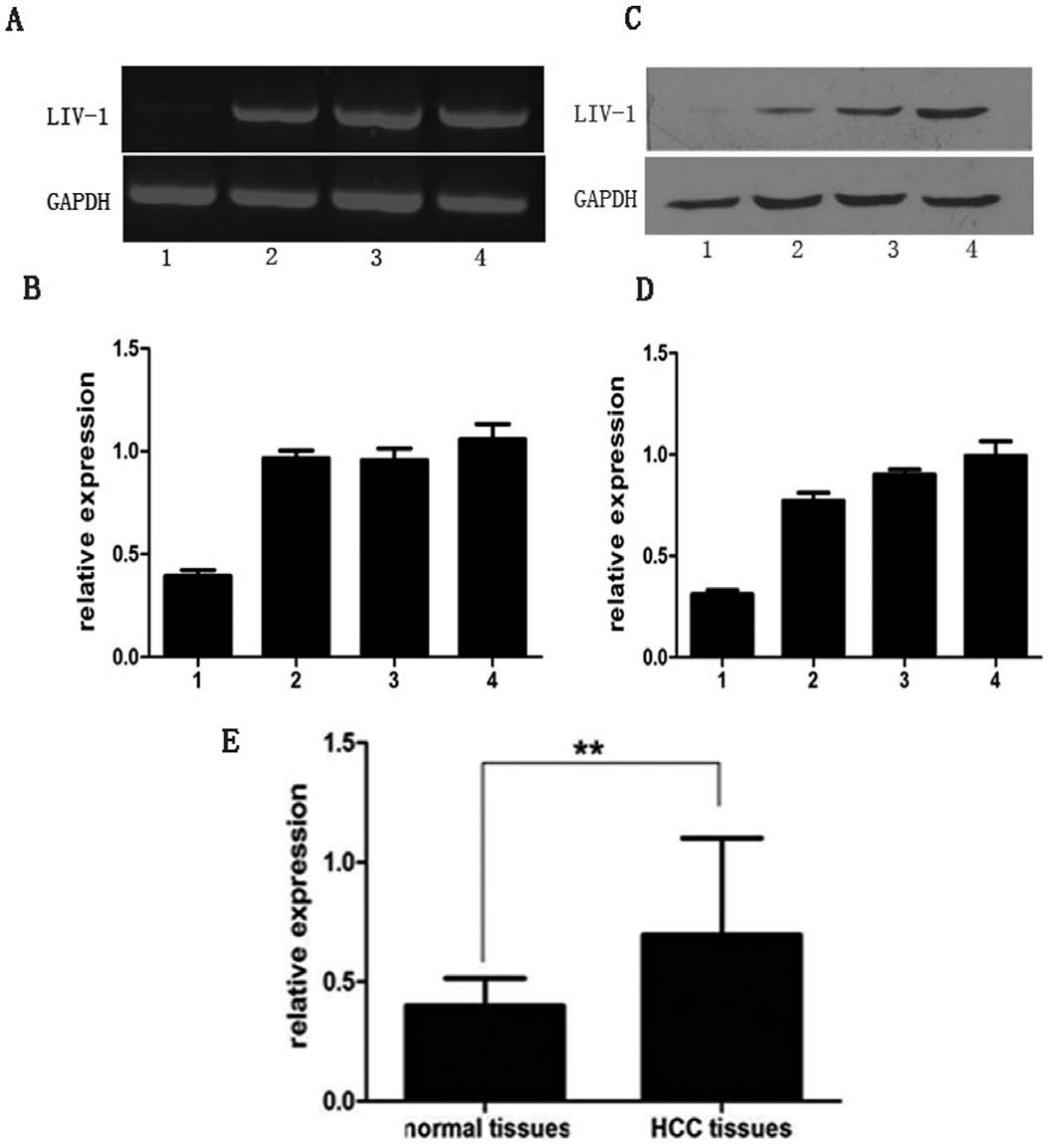
LIV-1 mRNA and protein expression levels in three cancer cell lines (SMMC-7721, Hep-3B, Hep-G2) and one normal liver cells line (L02) were investigated by RT-PCR (A and B) and Western blot (C and D). The relative expression levels were expressed as the ratio of band intensity for LIV-1 relative to that for GAPDH. The expression levels of these cell lines were more intense than that in L02 cells. (E) Western blot showed the higher LIV-1 expression in HCC tissues compared to normal liver tissues. **P<0.01.1: L02;2: SMMC-7721;3: Hep-3B;4: Hep-G2.

As shown in Fig. 1A and B, three liver cancer cell lines expressed higher levels of LIV-1 mRNA compared with L02 cells. Consistently, transcription level for LIV-1 was also higher in liver cancer cell lines compared with L02 cells (Fig. 1C and D). LIV-1 protein was higher in HCC tissues than that in normal tissues (0.694±0.402 vs. 0.398±0.117, respectively, *P*<0.01, Fig. 1E). The expressions of LIV-1 in 61 of 100 (61%) HCC tissues were higher than the mean level in normal tissues. And significant correlation was found between LIV-1 expression and tumor size (*P*<0.05) and lymph node metastases or microscopic vascular invasion (*P* = 0.037) (Table 1).The results between clinicopathological findings and LIV-1 are summarized in Table 1.

**Table 1:** Correlation between clinicopathological findings and LIV-1 expression.

LIV-1 protein level				
Factor	less thannormal	more thannormal		P-value
Age				
<60	28	44	0.971^b^	
≥60	11	17		
Sex				
Male	26	37	0.544^b^	
Female	13	24		
Lymph node metastases or microscopic vascular invasion				
Negative	21	20	0.037^b^	
Positive	18	41		
Tumor size				
<5 cm	22	22	0.046^b^	
≥5 cm	17	39		
Histologic differentiation				
Well	1	2	0.408^a^	
Moderate	25	46		
Poorly	13	13		
TNM stage				
Stage I	3	1	0.403^a^	
Stage II	16	23		
Stage III	20	34		
Stage IV	0	1		

a Analysed by Fisher’s exact test.

b Analysed **χ**2 test.

### LIV-1 Protein Location in HCC

Then we investigated the localization of LIV-1 protein in HCC tissues. Although LIV-1 was reported as a cell surface protein, we found LIV-1 expression was occasionally detected in cytoplasm of carcinoma cells.

### Inhibition of LIV-1 Expression by LIV-1 siRNA Alters Expression Level of E-cadherin

**Figure 2:**
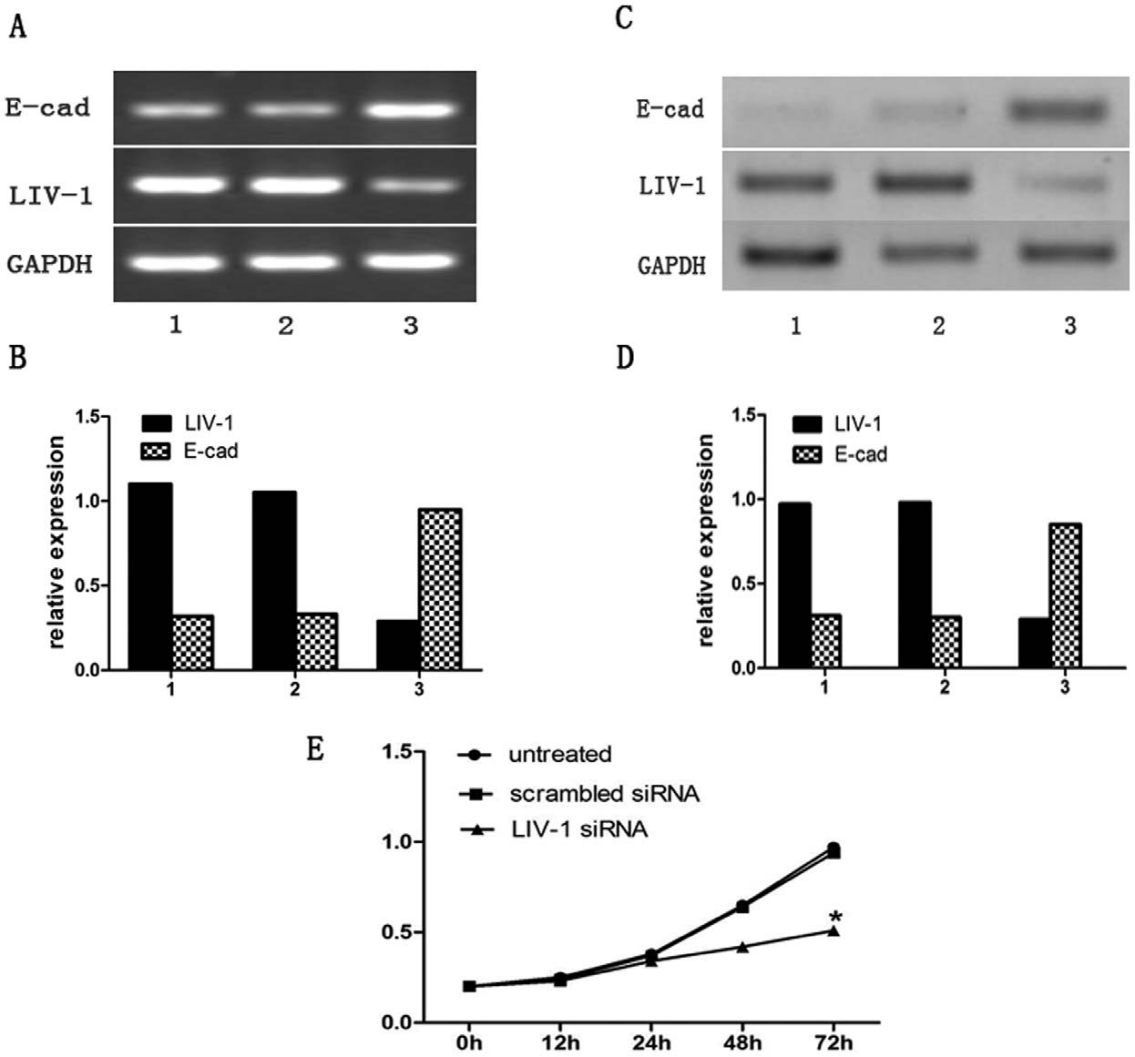
LIV-1 and E-cadherin expression in Hep-G2 cells after transfection with LIV-1 siRNA or scrambled siRNA. At 48 h post-transfection, the total RNA and protein were extracted and subjected to RT-PCR analysis (A and B) and Western blot (C and D). The relative mRNA and protein expression levels were expressed as the ratio of band intensity for LIV-1 relative to that for GAPDH. (E) Effect of silencing of LIV-1 on cell growth. At 0, 12, 24, 48 and 72 h post-transfection, the curve of cell growth was determined by MTT assay. Values were expressed as means ± SD, n = 6. *P<0.05 compared to control group.1: untreated; 2: scrambled siRNA; 3: LIV-1 siRNA.

We wondered if downregulation of LIV-1 led to change the level of expression of E-cadherin. As shown in Fig. 2, the mRNA (Fig. 2A and B) and protein expressions (Fig. 2C and D) of E-cadherin increased significantly in Hep-G2 cells transfected with LIV-1 siRNA. It implied LIV-1 might promote EMT by E-cadherin.

### Down-regulation of LIV-1 Suppressed Cell Growth of Hep-G2

**Figure 3:**
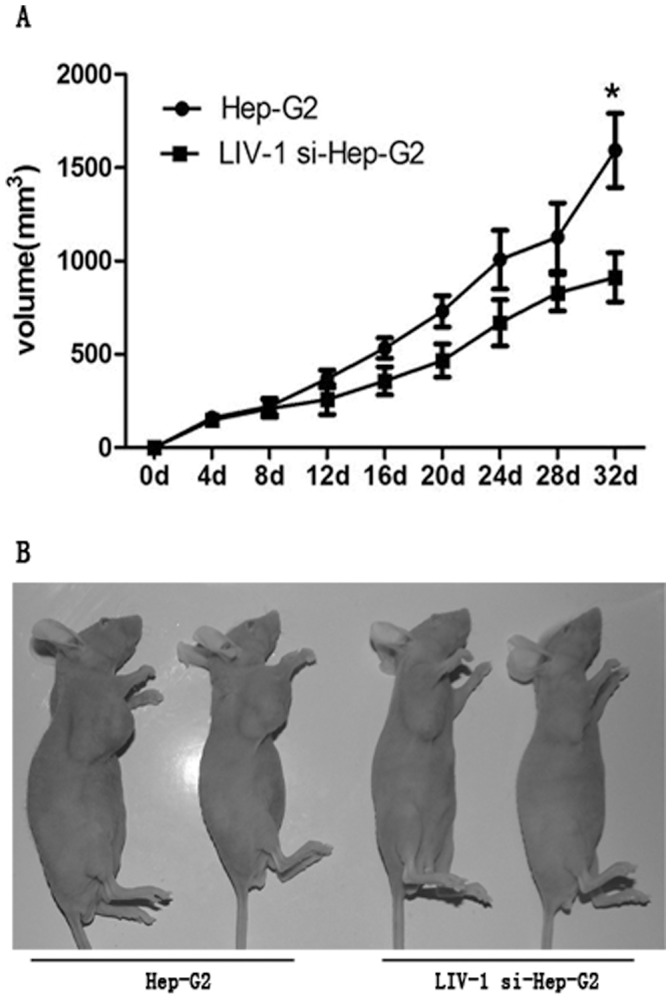
LIV-1 knockdown suppressed the tumor growth in nude mice. (A and B). Control (Hep-G2) cells (2×10^7^) or LIV-1si cells (LIV-1 siRNA Hep-G2) were injected subcutaneously into the right axillary cavity. After 32 days, the mice were sacrificed. LIV-1si cells exhibited significantly slower growth as well as smaller tumor size in nude mice compared to Hep-G2 cells. * *P*<0.05.

We next explored whether LIV-1 expression would affect the HCC cells growth. We examined the cell growth of LIV-1si Hep-G2 cells by MTT assay at 0, 12, 24, 48 and 72 h post-transfection. As shown in Fig. 3E, the results showed the inhibition effect at 72 h (*P<*0.05) after silencing of LIV-1, but scrambled siRNA cells did not change the cell growth significantly, which indicated that down-regulation of LIV-1 suppressed the growth of liver cancer cells.

### Reduced Expression of LIV-1 Inhibits Tumor Growth in Nude Mice

To determine whether LIV-1si also suppressed tumor growth in vivo, 2×10^7^ cells of Hep-G2 cells or LIV-1si Hep-G2 cells were injected subcutaneously into the axillary cavity of nude mice. We measured volume of tumor every four days after implantation.LIV-1si cells showed obvious slower growth and thus formed smaller tumors compared with those arising from Hep-G2 cells (Fig. 3A and B).

## Discussion

As we know, this is the first study to investigate the involvement of LIV-1 in human liver cancer. In present study, we observed results as following: 1) LIV-1 expression was more high in three liver cancer cell lines compared with normal liver cells.The transcription level of LIV-1 was higher in human liver cancer compared with normal tissues; 2) 61% liver carcinoma tissues expressed higher LIV-1 protein than the normal tissues and LIV-1 expression was associated with tumor size and lymphnode metastases or microscopic vascular invasion; 3) down-regulation of LIV-1 in liver cancer cells led to inhibition of cell growth both in vitro and in vivo.

In both cervical cancer and pancreatic cancer, the expression levels of LIV-1 were much higher in tumor tissues than in normal tissues [13], [14]; The knockdown of LIV-1 suppressed cell invasion and migration in cervical and pancreatic cancer cells. LIV-1 expression had also been reported to be promoted in clinical prostate cancer, which induced EMT in prostate cancer cells [15].Above of these indicated the tumor promoting effect of LIV-1. In contrast, LIV-1 protein level was associated negatively with tumor size, grade and stage in breast cancer tissues. In addition, expression levels of LIV-1 were associated with better outcomes in these patients [16].When it comes to why LIV-1 played various roles in the progression of different tumors, additional studies should be carried out in more other tumor tissues.

EMT was first recognized as a feature of embryogenesis in the early 1980s and found to participate in mesoderm and neural crest formation during normal development. More and more evidences supported EMT plays a critical role in the metastatic process of cancers [6], [7].

E-cadherin expression is a hallmark of EMT.E-cadherin expression is a dynamic process associated with the establishment of cell polarity and tissue organization. Reduced E-cadherin expression contributes to the transition of adenoma to carcinoma in animal models and is inversely correlated with tumor stage[17]–[19].The evidence that the expression of E-cadherin could be regulated by LIV-1 has also been reported in human breast cancer cell MCF-7 [20]. In our study, E-cadherin expression showed increased in LIV-1si cells, indicating that LIV-1 plays a vital role in EMT of liver cancer cells.

To demonstrate the function of LIV-1 as a regulator of EMT, our study showed that the reduction of LIV-1 expression inhibited cell and tumor growth in vitro and in vivo. LIV-1 expression was associated with tumor growth. It is possible that LIV-1 may lead liver cancer cells to more malignant phenotype through the induction of EMT [15].Although the molecular mechanisms need further studies, the present finding of significantly higher expression of LIV-1 in most carcinoma tissues also supports the idea that LIV-1 promotes the growth of cancer cells.

Taken together, our data showed that LIV-1 promoted EMT transition in HCC cells by regulating the expression of E-cadherin. We believe that our study is helpful to elucidate the precise role of LIV-1 in cancer progression. But in our study, only E-cadherin was examined whereas markers of EMT are more than E-cadherin. If more markers of EMT were detected, we will find a more exact relationship between LIV-1 and EMT, which is the focus of our next study.
